# Hysteroscopic Management of Complete Septate Uterus With Septate Cervix and Longitudinal Vaginal Septum in a Third-Level Mexican Institution: A Case Report

**DOI:** 10.7759/cureus.73384

**Published:** 2024-11-10

**Authors:** Bertha Patricia Díaz Sanginés, Humberto López Maldonado, Jahn Werner Von Der Meden Alarcón, Mayra Vallina Bocanegra, Lorena Matienzo Serment

**Affiliations:** 1 Gynecology, Centro Medico ABC, Mexico City, MEX; 2 Obstetrics and Gynecology, ABC Hospital, Santa Fe Campus, Mexico City, MEX

**Keywords:** asrm müllerian anomalies classification, complete septate uterus, dyspareunia, embryonic development, hysteroscopic treatment

## Abstract

Müllerian anomalies (MA) occur as a result of errors during embryogenesis. These changes are associated with genetic mutations, developmental disabilities, or environmental causes that affect the embryonic development stages. This paper describes a rare case report of a 22-year-old female who came to our hospital due to dyspareunia after one year of evolution. A thorough history, physical examination, and imaging studies led to the diagnosis of a complete septate uterus with septate cervix and longitudinal vaginal septum. Abdominal computed tomography diagnosed the left extrarenal pelvis. Hysteroscopic resection of the uterine septum and vaginal septoplasty were performed without any complications. Dyspareunia improved at the six-month follow-up. The description of this case is to share our experience and contribute to all common uncommon cases of MA reports.

## Introduction

Müllerian duct anomalies are composed of a spectrum of congenital anomalies affecting the development of the female reproductive tract. The incidence is estimated to be between 0.001% and 10% [1]. These anomalies can vary from malformations that go unnoticed to structural abnormalities that can directly impact style and quality of life. Among these, the combination of a complete septate uterus with a septate cervix and longitudinal vaginal septum is known to be a complex anomaly whose incidence cannot be estimated because of the lack of cases described in the literature [2]. 

During the seventh week of female embryonic development, the absence of the Müllerian inhibitory factor triggers the regression of the mesonephric ducts and stimulates the development of two paramesonephric ducts. These paramesonephric ducts eventually form the upper two-thirds of the vagina, cervix, uterus, and fallopian tubes [3]. By the eighth week, a septum is formed between the two paramesonephric ducts; this septum will reabsorb by the 20th week; if that doesn’t happen, the uterus will remain with a septum. This septum may be partial (from the uterine fundus without reaching the internal os) or may be complete, extending the cervix [4]. Furthermore, this septum can reach the vagina, extending itself vertically and dividing it into two separate compartments.

The main clinical manifestations are recurrent pregnancy loss, dyspareunia, dysmenorrhea, or even infertility [5]. Concomitant extragenital malformations can include the urinary system and skeletal system, such as renal agenesis and scoliosis [6]. 

Diagnosis often includes a combination of several imaging modalities like transvaginal ultrasound, magnetic resonance imaging (MRI), and hysteroscopy to accurately describe the particularities of each anomaly [7]. Treatment equally depends on the symptoms and reproductive goals of each patient. Surgical techniques (hysteroscopy or laparoscopy) seek to correct anatomical defects and improve fertility, but we should not forget that counseling and patient education are important steps of management [7]. 

## Case presentation

**Figure 1:**
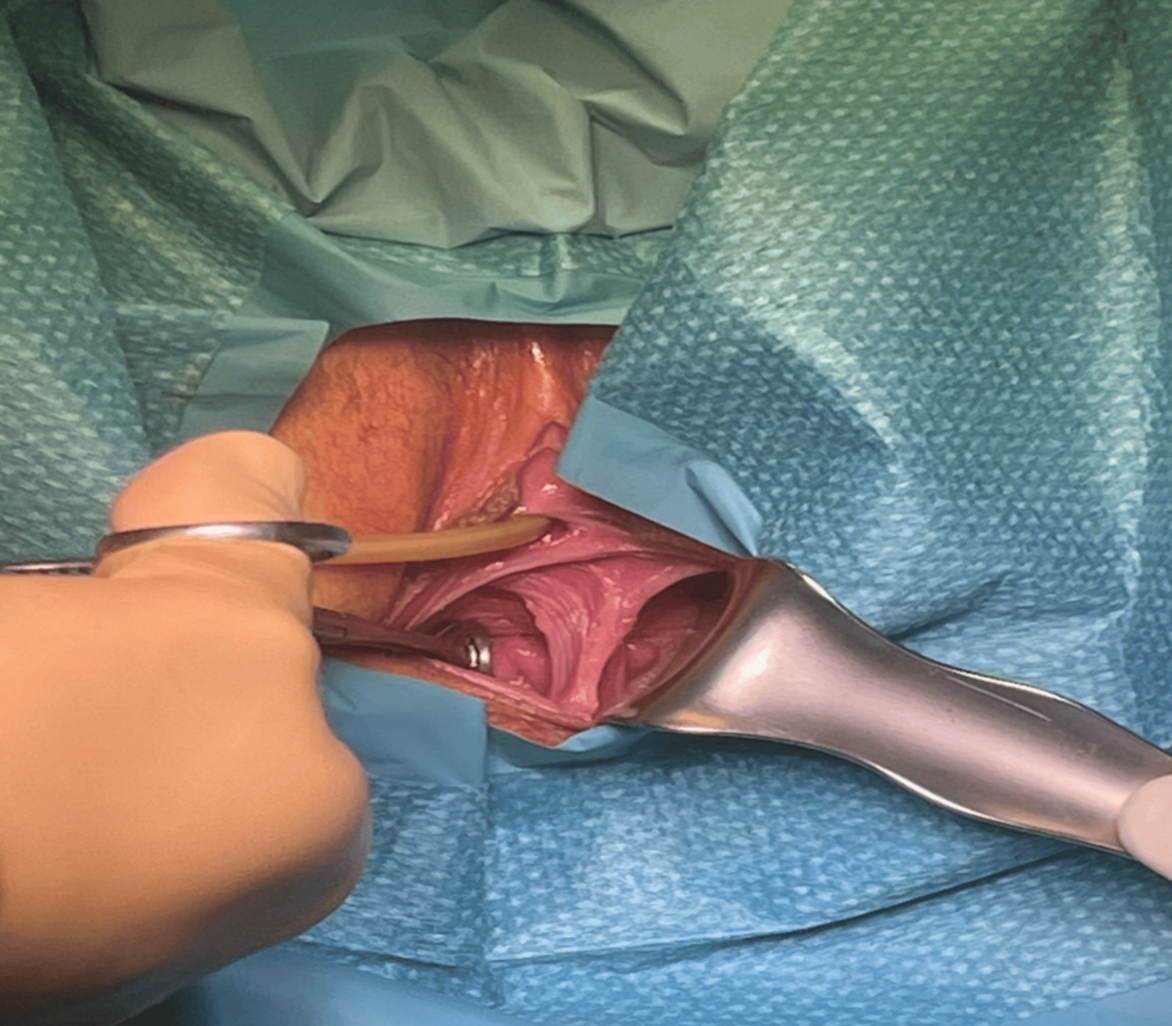
Longitudinal vaginal septum

A 22-year-old presented to our service complaining of dyspareunia associated with penetration disorder. She has a history of sluggish bowel syndrome and allergic rhinitis well controlled by treatment. Surgical history only reported amygdalectomy with no complications; no other significant past medical or surgical history was noted. Nulligravida, menarche at the age of 14, with regular menses every 28 days, lasting for six days, associated with dysmenorrhea, no history of oral contraceptive use. The patient´s family history was negative for any known anomaly. Pelvic examination revealed normal external genitalia, with the presence of a longitudinal vaginal septum and two uterine cervices (Figure 1).

**Figure 2:**
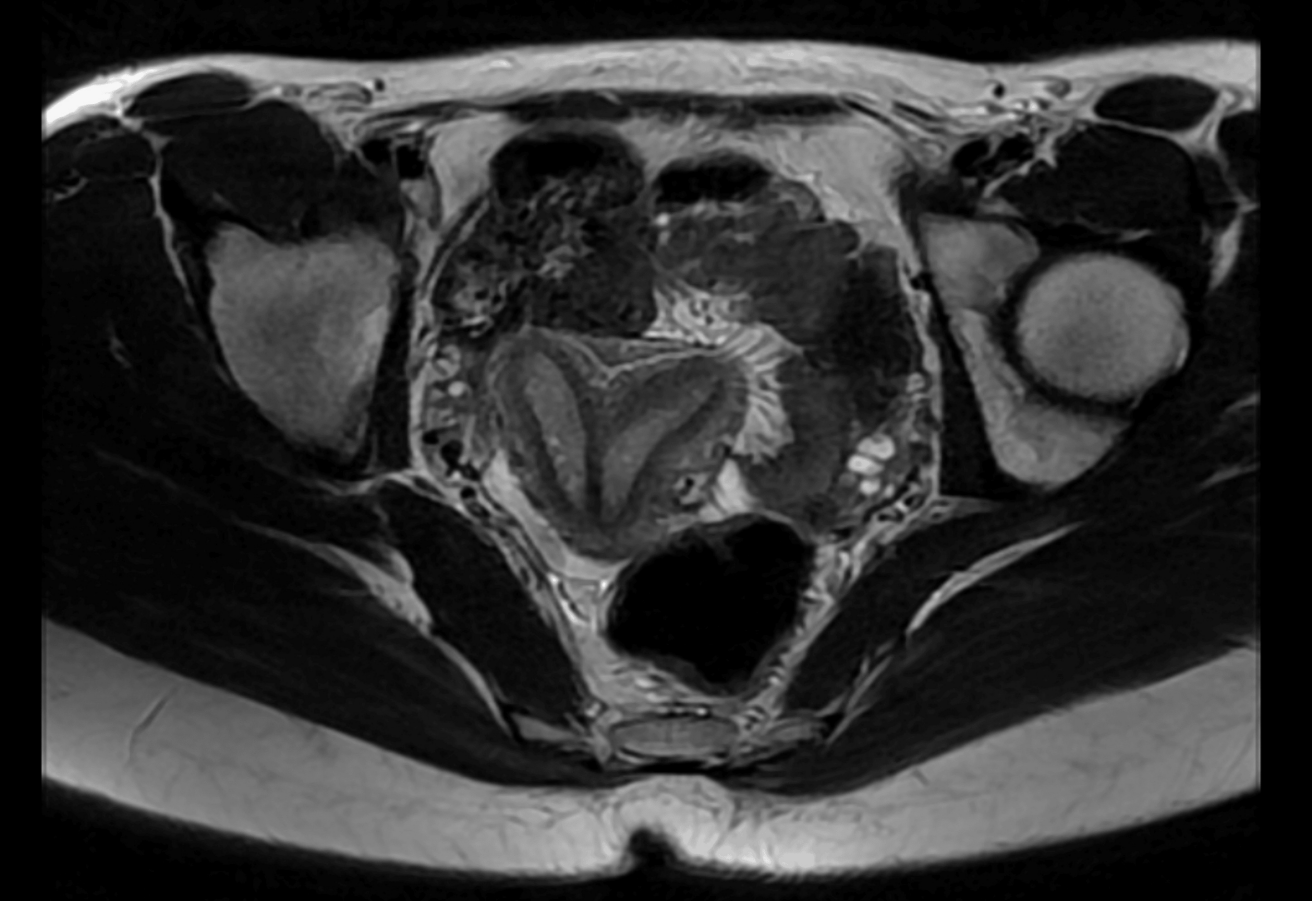
Complete septate uterus (MRI) MRI: magnetic resonance imaging

The MRI showed the presence of a uterus cavity with a complete septate uterus (Figure 2).

**Figure 3:**
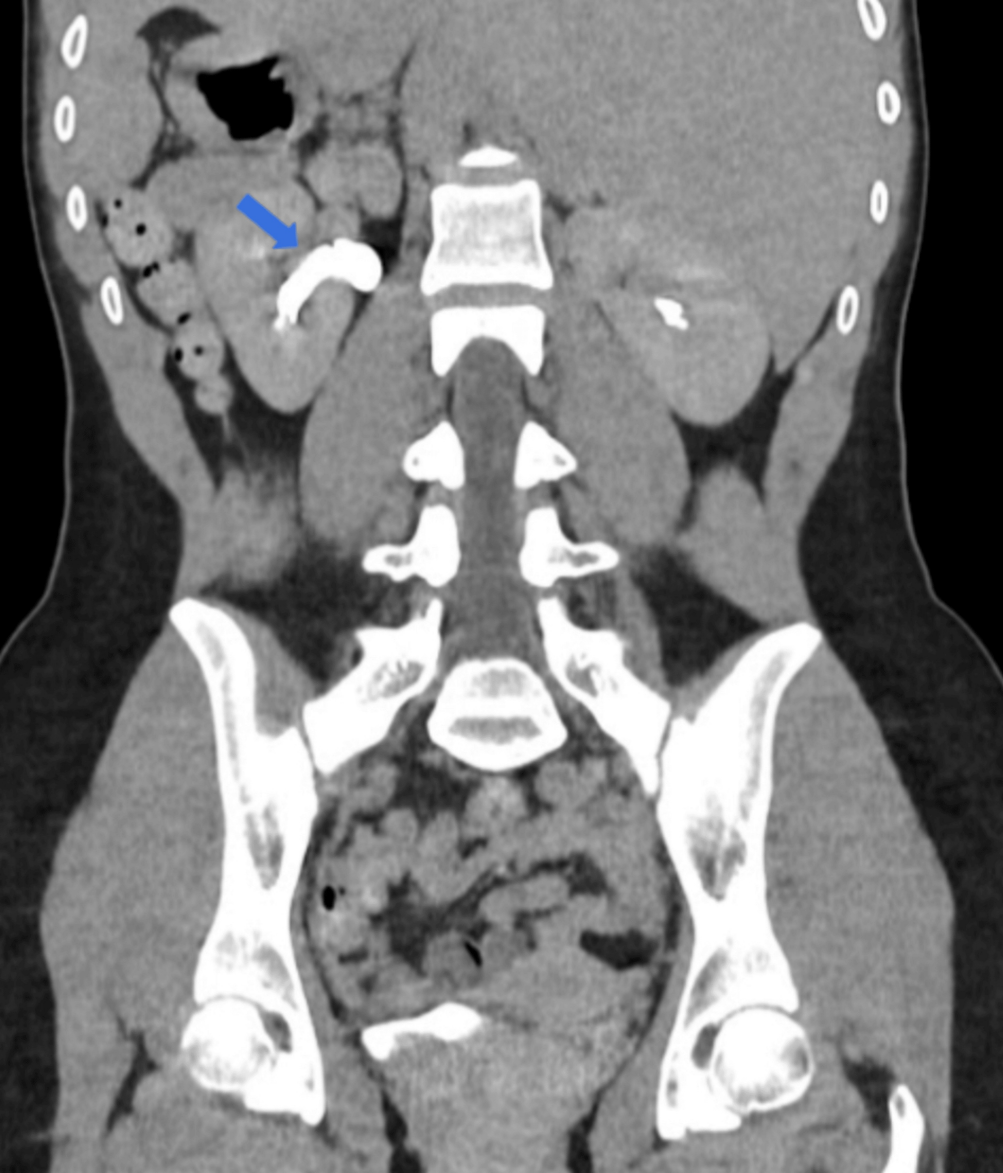
Left extrarenal pelvis (abdominal computed tomography)

The left extrarenal pelvis as an anatomical variant was diagnosed by abdominal computed tomography (Figure 3).

The patient was scheduled for resection of the vaginal and uterine septum by hysteroscopy under laparoscopic control.

**Figure 4:**
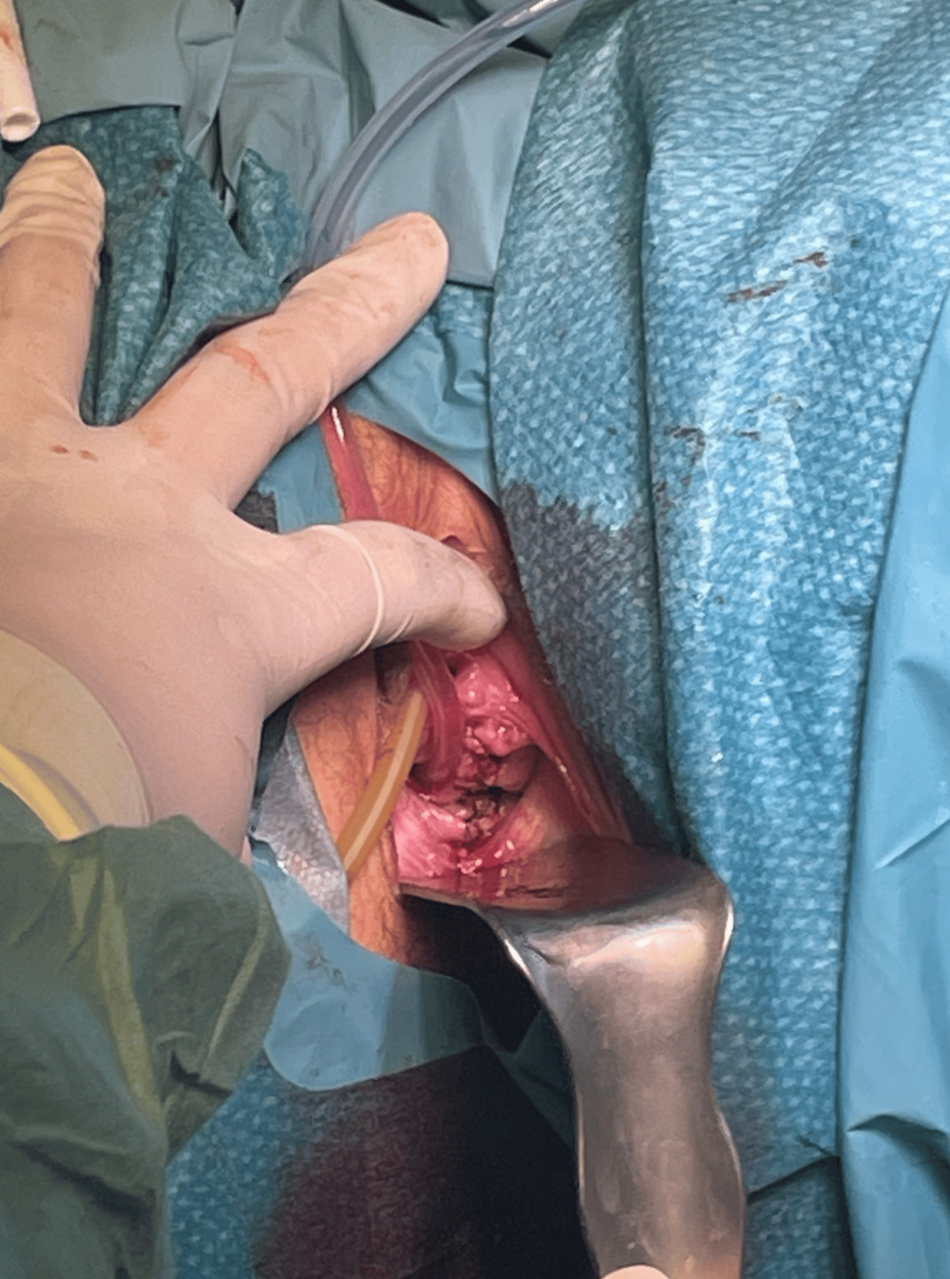
Vaginal septum resected

The resection of the vaginal septum was made with the patient in the dorsal lithotomy position and an adequate vaginal retractor to allow exposure to the septum. The septum was grasped with Allis clamps, and a horizontal incision was made through the septum; the edges were sutured with 3-0 vicryl (Figure 4). 

**Figure 5:**
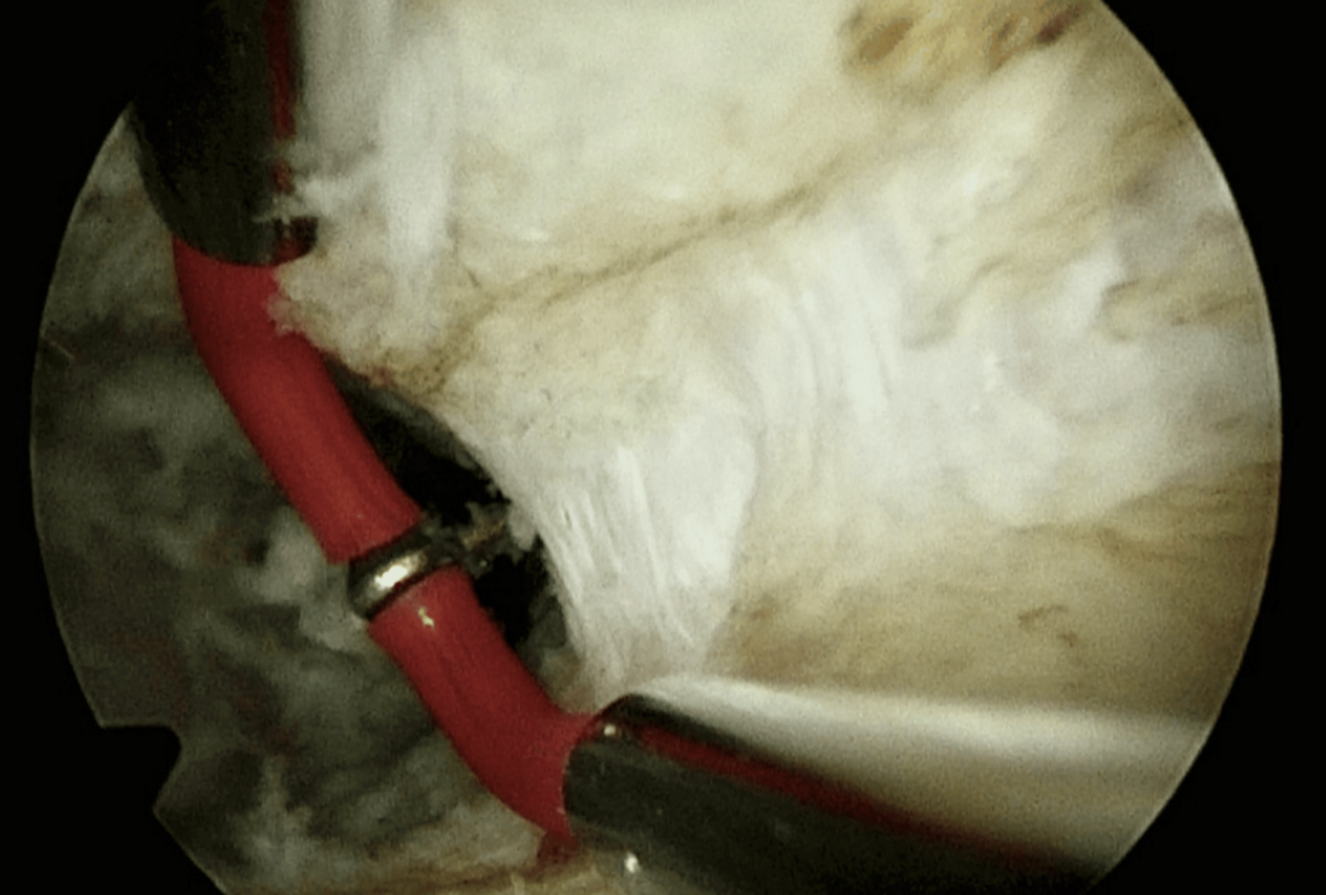
Hysteroscopic metroplasty

Hysteroscopic metroplasty was performed with bipolar energy in a distal to the proximal direction (Figure 5) until the two cavities communicated, all the time under laparoscopic control to prevent uterine perforation. At the end of the procedure, 6 ml of hyaluronic acid (8 mg/ml) was placed through the external cervical os to prevent the formation of intrauterine adhesions. The patient was discharged in 24 hours without pain. 

## Discussion

MA are developmental anomalies of the female reproductive tract. The American Fertility Society (AFS) classification from 1988 is the most recognized and utilized among the numerous proposed classification systems for Müllerian anomalies. Vaginal and cervix malformations are excluded from the AFS classification. The wide range of MA is still largely unknown and confusing, despite the numerous classifications. The American Society of Reproductive Medicine (ASMR) Müllerian Anomalies Classification 2021 (MAC 2021) classifies Müllerian anomalies (MA) into nine categories (müllerian agenesis, cervical agenesis, unicornuate uterus, uterus didelphys, bicornuate uterus, septate uterus, longitudinal vaginal septum, transverse vaginal septum, complete anomalies) [8].

**Figure 6:**
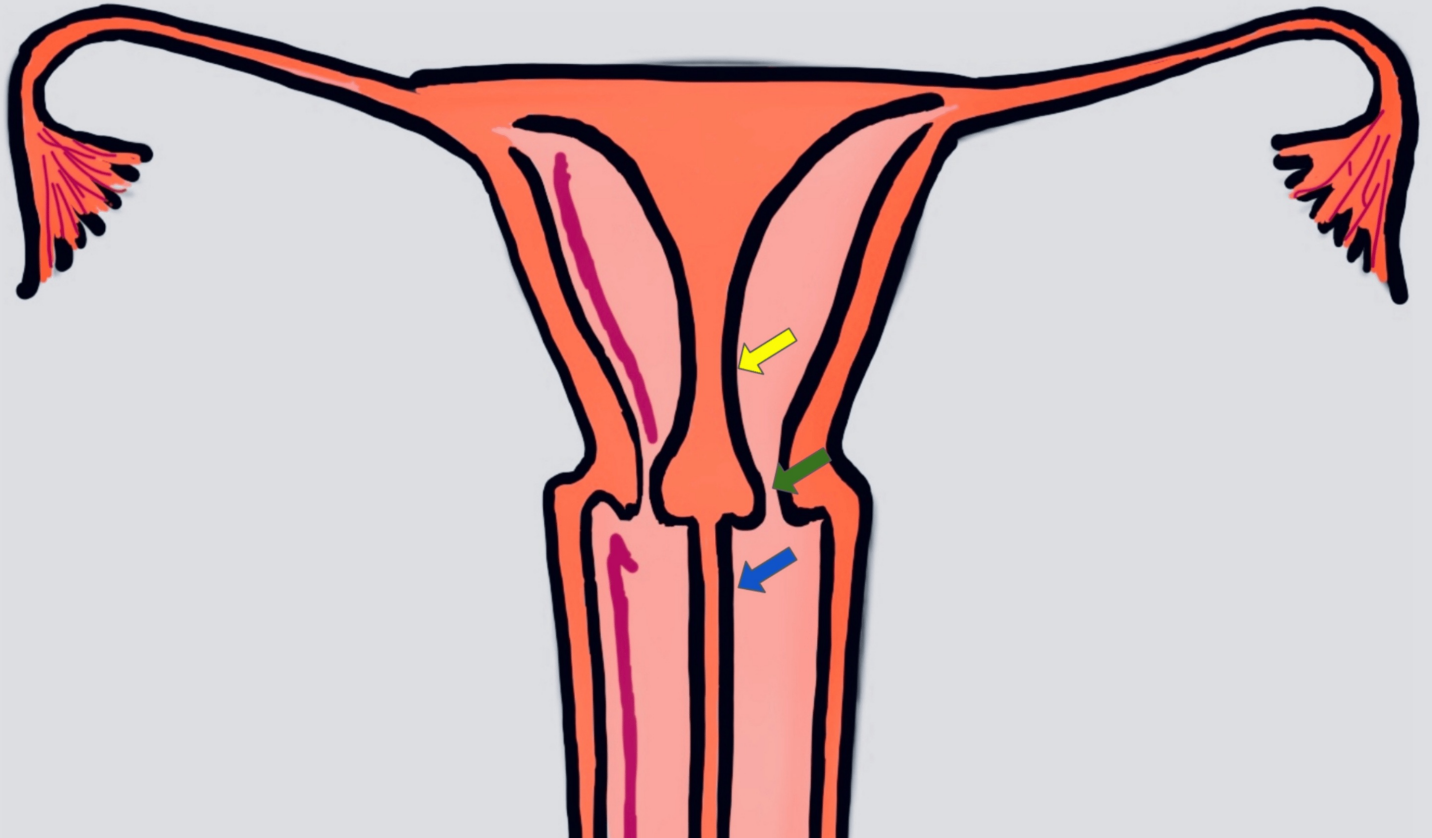
Complete septate uterus (yellow arrow) with septate cervix (green arrow) and longitudinal vaginal septum (blue arrow)

Our patient falls into the category of the septate uterus (Figure 6); this clinical case report does not aim to describe all Müllerian anomalies.

Anatomical disorders of the female reproductive system occur frequently, ranging from congenital absence of the vagina or uterus to fusion defects of the Müllerian ducts (MD). Environmental causes and genetic mutations that affect the embryonic development stages are associated with these changes [9].

Five weeks into pregnancy, the genital tracts begin to form and are fully formed between 16 and 20 weeks. An alternative bidirectional theory and a unidirectional regression theory are the two theories that explain the evolution of MA. According to the bidirectional theory, the process proceeds simultaneously in the cranial and caudal directions, beginning from the uterine isthmus. The bidirectional theory can explain the MA with a complete septum, a duplicate cervix, and a transverse vaginal septum [10]. 

A complete septate uterus with cervical duplication and longitudinal vaginal septum is an uncommon uterine malformation. A complete longitudinal uterine septum is usually associated with a complete vaginal septum, which divides both uterine cavities and cervical canals. This malformation is associated with a high rate of infertility, recurrent pregnancy loss, preterm delivery, dysmenorrhea, and dyspareunia. Therefore, resections of the uterine and vaginal septum are generally recommended [11]. 

Up to 30% of cases are associated with urinary tract malformations, so it should always be evaluated by imaging studies such as an MRI or CT scan [12].

MRI is the modality of choice to diagnose urogenital anomalies. It has been shown to correctly determine the type of Müllerian anomaly in 96% of patients and can help determine the exact nature of MA present before surgery. The use of 3-D ultrasound in diagnosis has recently been found to be comparable to MRI and more economical [1]. 

Hysteroscopic metroplasty is the gold standard for the treatment of the septate uterus, and it has become a routine practice for hysteroscopists [13]. 

## Conclusions

Müllerian duct anomalies are a group of malformations whose symptoms can vary widely depending on the present defect. These symptoms can range from uterine bleeding disorders and infertility to malformations in the genitourinary system. It is a challenge to the physician as well as to the patient to undergo the process of re-establishing the anatomy to solve the clinical manifestations. This process will probably need a surgical procedure by hysteroscopy or laparoscopy, but counseling and education should not be forgotten. 

The patient mentioned in this case report arrived to eliminate the pain she experienced during intercourse. Physical exploration was performed, finding a longitudinal vaginal septum associated with two cervices. Due to these findings, an MRI was requested, which showed the presence of a complete septate uterus with no other system malformations. The resection of the vaginal septum was accomplished, as was a hysteroscopic metroplasty under laparoscopic control and ending with intrauterine hyaluronic acid. No complications were presented, and the patient was discharged. 

## References

[1] Louden ED, Awonuga AO, Gago LA, Singh M (2015). Rare Müllerian anomaly: Complete septate uterus with simultaneous longitudinal and transverse vaginal septa. J Pediatr Adolesc Gynecol.

[2] Pozzati F, Mirandola M, Topozouva G, Parodi L, Carla Testa A, Scambia G, Catena U (2023). Complete uterine septum, double cervix, and longitudinal vaginal septum: an integrated approach for one-stop diagnosis and ultrasound-guided endoscopic treatment. Facts, Views & Vision.

[3] Passos IM, Britto RL (2020). Diagnosis and treatment of müllerian malformations. Taiwan J Obstet Gynecol.

[4] Blanco-Breindel MF, Kahn J, Singh M (2024). Septate Uterus. StatPearls [Internet].

[5] Santana González L, Artibani M, Ahmed AA (2021). Studying Müllerian duct anomalies - from cataloguing phenotypes to discovering causation. Dis Model Mech.

[6] Su S, Bao XM, Wang S (2024). Concomitant extragenital malformations of female reproductive tract anomalies: analysis of 444 cases in Peking Union Medical College Hospital (Article in Chinese). Zhonghua Fu Chan Ke Za Zhi.

[7] Committee on Adolescent Health Care (2018). ACOG Committee Opinion No. 728: Müllerian agenesis: diagnosis, management, and treatment. Obstet Gynecol.

[8] The American Fertility Society (1988). The American Fertility Society classifications of adnexal adhesions, distal tubal occlusion, tubal occlusion secondary to tubal ligation, tubal pregnancies, müllerian anomalies and intrauterine adhesions. Fertil Steril.

[9] Sugiura-Ogasawara M, Ozaki Y, Suzumori N (2013). Müllerian anomalies and recurrent miscarriage. Curr Opin Obstet Gynecol.

[10] Muller P, Musset R, Netter A, Solal R, Vinourd JC, Gillet JY (1967). State of the upper urinary tract in patients with uterine malformations. Study of 133 cases (Article in French). Presse Med (1893).

[11] Patton PE, Novy MJ, Lee DM, Hickok LR (2004). The diagnosis and reproductive outcome after surgical treatment of the complete septate uterus, duplicated cervix and vaginal septum. Am J Obstet Gynecol.

[12] Robbins JB, Broadwell C, Chow LC, Parry JP, Sadowski EA (2015). Müllerian duct anomalies: embryological development, classification, and MRI assessment. J Magn Reson Imaging.

[13] Yang JH, Chen MJ, Shih JC, Chen CD, Chen SU, Yang YS (2014). Light-guided hysteroscopic resection of complete septate uterus with preservation of duplicated cervix. J Minim Invasive Gynecol.

